# Déjà-vu? Neural and behavioural effects of the 5-HT_4_ receptor agonist, prucalopride, in a hippocampal-dependent memory task

**DOI:** 10.1038/s41398-021-01568-4

**Published:** 2021-10-04

**Authors:** Angharad N. de Cates, Lucy C. Wright, Marieke A. G. Martens, Daisy Gibson, Cagdas Türkmen, Nicola Filippini, Philip J. Cowen, Catherine J. Harmer, Susannah E. Murphy

**Affiliations:** 1 Department of Psychiatry, University of Oxford, Warneford Hospital, Oxford, UK; 2 Oxford Health NHS Foundation Trust, Warneford Hospital, Oxford, UK; 3 Department of Neuropsychology and Psychopharmacology, Faculty of Psychology and Neuroscience, Maastricht University, Maastricht, The Netherlands; 4 IRCCS San Camillo Hospital, Venice, Italy; 5 Oxford Centre for Human Brain Activity, Wellcome Centre for Integrative Neuroimaging, Department of Psychiatry, University of Oxford, Oxford, UK

**Keywords:** Hippocampus, Clinical pharmacology

## Abstract

Cognitive deficits commonly accompany psychiatric disorders but are often underrecognised, and difficult to treat. The 5-HT_4_ receptor is a promising potential treatment target for cognitive impairment because in animal studies 5-HT_4_ receptor agonists enhance hippocampal-dependent memory processes. To date, there has been little work translating these effects to humans. We tested whether short-term administration of the 5-HT_4_ partial agonist, prucalopride, modified behavioural and neural (fMRI) memory processing in 44 healthy human volunteers using an experimental medicine model. We found that participants who had received six days of prucalopride treatment were significantly better at recalling previously seen neutral images and distinguishing them from new images. At a neural level, prucalopride bilaterally increased hippocampal activity and activity in the right angular gyrus compared with placebo. Taken together, these findings demonstrate the potential of 5-HT_4_-receptor activation for cognitive enhancement in humans, and support the potential of this receptor as a treatment target for cognitive impairment.

## Introduction

Impairments in cognitive function are present across the spectrum of psychiatric disorders, including affective, psychotic, and neurodevelopmental conditions [1, 2]. These cognitive deficits may appear alongside but seem separate from other coexisting psychopathology, and often are not well treated by interventions that relieve other symptoms of the disorder [2]. This may in part relate to their breadth: cognitive dysfunction is not limited to memory deficits, but also includes impairments in learning, reward, attention, motivation, and language, as well as processing speed [2]. Given the significant impact such impairments can have on quality of life, there is a critical need for the development of treatments that can ameliorate the cognitive deficits associated with psychiatric disorder. While there are some interesting pharmacological targets identified in animal studies, there is an outstanding need to translate this work into humans.

Preclinical studies have demonstrated that the serotonin_4_ (5-HT_4_) receptor is a promising treatment target for cognitive impairment. 5-HT_4_ is an excitatory G-protein-coupled postsynaptic receptor expressed in brain areas involved in emotion and cognition, such as the basal ganglia, hippocampus, amygdala, and prefrontal cortex [3–7]. Following 5-HT_4_-receptor agonist administration, rodents demonstrate improved performance during learning and memory tasks [8, 9], with effects obvious after only a few doses [10, 11]. This is particularly apparent in tasks tapping hippocampal-dependent learning and memory [9], which likely relates to various downstream effects of 5-HT_4_-receptor activation including increased hippocampal cell proliferation and promotion of learning-induced spine growth [10, 12, 13], induction of long-term potentiation (LTP) in the hippocampus [14], increased release of neurotransmitters such as acetylcholine in the hippocampus and frontal cortex [9, 15], and increased expression of neuroplasticity proteins such as brain-derived neurotrophic factor (BDNF) [9, 10, 12]. Cortical release of acetylcholine also appears to increase the power of hippocampal θ oscillations following 5-HT_4_-receptor agonism; these oscillations have been linked to memory and attentional performance in both animal and human studies [16]. Agonism at the 5-HT_4_ receptor also modulates the release of other neurotransmitters relevant for cognition, including GABA [17], glutamate [18], dopamine [19], and histamine [16]. The importance of 5-HT_4_-receptor agonism and the downstream increase in acetylcholine and change in other neurotransmitters for cognition is further demonstrated by two key preclinical findings. First, 5-HT_4_ agonist-induced pro-cognitive effects can be blocked by the coadministration of 5-HT_4_-receptor antagonists [11, 20–22]. Second, anticholinergic-induced cognitive impairments can be reversed by the administration of 5-HT_4_-receptor agonists [20, 22–25].

Given the evidence from animal models, 5-HT_4_-receptor agonism was initially considered as a potential therapeutic option for primary dementias [26–28]. However, investigating this mechanism in humans has been limited due to the side-effect profile of early agents. Prucalopride, a selective high-affinity 5-HT_4_ partial agonist with good brain penetration [16], recently received a medical license for constipation. This has allowed us to investigate 5-HT_4_-receptor agonism in humans using an experimental medicine model to assess potential enhancement of cognition in healthy humans. We have previously demonstrated that a single 1-mg dose of prucalopride has pro-cognitive effects across three different tasks of learning and memory (the Rey Auditory Verbal Learning Task (RAVLT), the Probabilistic Instrumental Learning Task (PILT), and emotional memory as part of the Emotional Test Battery (ETB)) [29]. Here we examine the effect of a longer period of prucalopride administration (six days at testing) on behavioural and neural learning and memory processes, including a memory paradigm known to be a reliable probe of hippocampal function and related circuitry [30, 31]. We hypothesised that prucalopride would improve episodic memory and increase the activation of the hippocampus and related neural circuitry during memory retrieval.

## Materials/subjects and methods

### Participants

In total, 44 right-handed healthy participants (21:23 = placebo: prucalopride), between 18 and 36 years, were administered either prucalopride (seven days × 1 mg (imaging occurring on day 6)) or placebo, in a double-blind, randomised design. Participants were fluent in English and were screened for contraindications to prucalopride. Inclusion and exclusion criteria are included in detail in Supplementary Material; in brief, participants were young adults of either sex, healthy, right-handed, not pregnant or breast feeding, and with a BMI between 18 and 30. We also excluded those who may be more vulnerable to the side effects of prucalopride. The study was approved by the University of Oxford Central University Research Ethics Committee (MSD-IDREC reference R57219/RE001) and a protocol including outcomes was preregistered with clinicaltrials.gov (NCT03572790). The results detailed here relate to predefined secondary outcomes. No changes to the methods occurred after the start of the study. Participants gave written informed consent.

### Design and randomisation

The study had a between-subject, double-blind, placebo-controlled design. Participants were randomly assigned to seven days of prucalopride 1 mg (Resolor) or placebo (lactose tablets, Rayonex Biomedical) in a 1:1 allocation. Randomisation was blocked in design (block size = 4) and performed using an online tool (sealedenvelope.com on 1^st^ June 2018). Drug allocation was stratified for sex. Allocation was concealed from participants, investigators, and assessors using sequential-numbered containers, and the encapsulation process ensured that both capsules appeared identical. All capsules were consumed by participants in their own home.

Previous studies examining the effect of prucalopride and other serotonergic drugs on neurocognitive outcomes, including learning/memory and emotional processing, indicate that an effect size of 0.8–0.9 may be expected [32, 29, 32]. However, given how little is known about the effects of 5-HT_4_ receptors on cognition in humans, we took a conservative approach and calculated the samples size needed on the basis of a smaller estimated effect size (0.5–0.7), indicating that 17 participants per group are needed to give 90% power to detect a significant difference between the two groups with an α of 5%. We therefore aimed to recruit 50 (25 in each group) as part of oversampling and to ensure adequate power for fMRI analyses.

By day 6 when imaging was carried out, prucalopride would be expected to be at a steady state (terminal half-life approximately 24 h) [33]. Testing occurred at the Department of Psychiatry and the Oxford Centre for Human Brain Activity (OHBA), part of the Wellcome Integrative Neuroimaging Centre (WIN). It was avoided during the premenstrual week for female participants.

### Questionnaire measures

Participants completed the following self-report questionnaires to obtain baseline measures of mood, anxiety, and personality: Beck Depression Inventory-II [34], Snaith–Hamilton Pleasure Scale (SHAPS) [35], Spielberger State-Trait Anxiety Inventory, Trait Version (STAI-T) [36], and Eysenck Personality Questionnaire [37]. Affect and anxiety were measured at three times (baseline, preimaging (day 6), and post imaging (day 6)): affect using the Positive and Negative Affect Scale (PANAS) [38] and the visual analogue scale (VAS) [39], and for anxiety the Spielberger State-Trait Anxiety Inventory, State Version (STAI-S) [36]. Side effects were measured at the same three time points using a scale where participants rated the extent to which they were experiencing each of the most commonly reported side effects of prucalopride [40]. At the end of the study (day 7), participants guessed their drug allocation with a forced-choice question.

### Memory encoding task

**Fig. 1:**
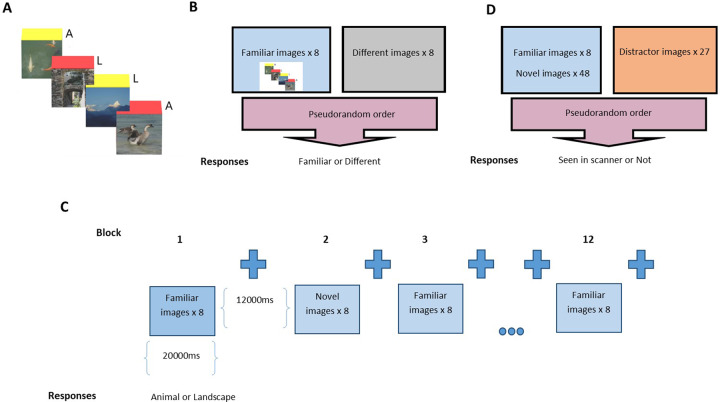
The memory-encoding task design, including fMRI memory-encoding training of familiar images for all participants. **A** Before and outside the scan, eight randomly selected “familiar” pictures (four animals (A), four landscapes (L)—4 examples shown) were presented eight times in a pseudorandom order on a computer (PC) screen [image presentation 3250 ms, interstimulus interval 500 ms, and between-block interval (fixation cross) 5000 ms]. Participants identified these as animal/nonanimal using a 2-button response. **B** Immediately before the scan, participants underwent a prescan recall test where they were asked to identify which images were familiar from (A) and which were different. **C** During the scan, familiar and novel images were presented as follows: image presentation 2000 ms, interstimulus interval 500 ms, and block duration 20000 ms. There were six familiar and six novel image blocks. Between each block of images, there was 12000 ms of rest, during which subjects passively viewed a fixation cross (a total of 12 rest blocks). Subjects were instructed to select from a 2-button response according to whether the images were animal/nonanimal. Responses were monitored by the scanner operators to ensure compliance and accuracy and were registered by the software in a text file. **D** After the scan, participants took part in a post-scan recall test where they were asked to identify which images had been seen in the scanning task (C) and which were distractors.

On the 6^th^ day of prucalopride administration, participants underwent a 3T scan, including an fMRI memory task designed to induce hippocampal activation. In this task (adapted from [30, 31]), participants were presented with coloured emotionally neutral images of animals or landscapes similar in complexity and brightness. Before the scan, randomly preselected pictures (four animals, four landscapes) were presented eight times in a pseudorandom order on a screen, called the “familiar” images (see Fig. 1(A)). Participants were asked to identify these as animal/nonanimal using a two-button response. To ensure that participants had satisfactorily encoded these images before the scan, a memory task was performed (eight “familiar” plus eight different images) involving the same number of animals and landscapes presented in pseudorandom order, where subjects were asked to identify these as familiar or different using a two-button response (see Fig. 1(B)).

During the scan, images were displayed in a pseudorandom order in a blocked design with 12 task blocks that were interleaved with rest blocks (during which participants were required to fixate on a cross on the screen) (see Fig. 1(C)). In each task block, eight images were presented in a pseudorandom order: either eight “familiar” images (the images presented prescan) or eight “novel” images (previously unseen images). Familiar images were presented in a different order in each block. Novel images were taken from a pool of 48 images (24 animals and 24 landscapes) and each was only presented once during the task. Participants were required to indicate via a two-button response if the image contained an animal or not. Participants were also instructed to try to remember the images for a subsequent memory task. After the scan, 83 images were presented on a PC screen for 4000 ms each (interstimulus interval 1000 ms): the eight “familiar” images (seen prescan and during scan), the 48 “novel” images (seen during scan only), and 27 “distractors” (images never seen before: 13 animals and 14 landscapes) were displayed in pseudorandom order (see Fig. 1(D)). Participants were required to indicate via a button press whether the images had been seen inside the scanner or not. The task was programmed in Presentation (Neurobehavioral Systems; https://www.neurobs.com).

This task was designed to stimulate implicit visual memory recognition in the scanner for images that participants had seen before (familiar images), compared to images that were new to participants in the scanner (novel images) where implicit encoding should occur.

### Demographic and behavioural data analysis

Demographic characteristics and baseline clinical measures were analysed using independent-sample *t*-tests (continuous variables), Fisher’s exact tests (sex and native language), and logistic regression (education). A repeated-measures analysis of variance (ANOVA) was used to analyse group differences in self-report measures and behavioural performance in the fMRI experimental task. Levene’s test (*t-*tests) and the Greenhouse–Geisser procedure (ANOVAs) were used where appropriate. A *p*-value less than 0.05 was used to denote statistical significance. Partial eta squared is reported as a measure of effect size. Behavioural data were analysed in SPSS (version 25, IBM). Graphs were produced using GraphPad Prism (version 9) and Excel (version 2016). Data were checked to ensure that tests and procedures were valid as appropriate.

### MRI data acquisition and analysis

Blood-oxygenation-level-dependent (BOLD) fMRI and T1-weighted anatomical images were acquired using a 3-Tesla Siemens Prisma scanner, equipped with a 32-channel head matrix coil (Siemens, Erlangen, Germany). While in the scanner, participants also completed an emotional face task and resting-state scan (reported elsewhere), and an arterial spin-labelling (ASL) scan, which is reported in brief here. Foam padding and a head restraint were used to control head movement. Further details of fMRI and structural MRI acquisition can be found in the Supplementary Material.

Imaging data were analysed with FSL (www.fmrib.ox.ac.uk/fsl). fMRI data were preprocessed and analysed using FEAT (FMRI Expert Analysis Tool), version 6.0.4, part of FSL (FMRIB’s Software Library; www.fmrib.ox.ac.uk/fsl). For more information on the steps involved in data preprocessing, first-level and second-level analyses, and confirmatory analyses, please refer to the Supplementary Material.

In the first-level analysis, individual activation maps were computed using the general linear model with local autocorrelation correction. Two explanatory variables were modelled: “novel” and “familiar” images. Temporal derivatives were included in the model. Variables were modelled by convolving each block with a haemodynamic response function, using a variant of a gamma function (i.e., a normalisation of the probability-density function of the gamma function) with a standard deviation of 3 s and a mean lag of 6 s. No included participant demonstrated significant movement: absolute displacements were less than one voxel and relative displacements less than ½ voxel. At the whole-brain level, familiar images were contrasted with novel, resulting in the following model: (1) novel vs. baseline; (2) familiar vs. baseline; (3) novel > familiar; (4) novel < familiar.

In the second-level analysis, whole-brain individual data were combined at a group level (participants on placebo vs. prucalopride) using a mixed-effect analysis, and cerebral blood flow and grey matter maps as covariates of no interest. Groups were contrasted with each other, resulting in the following comparisons: (1) placebo > prucalopride, (2) prucalopride > placebo, (3) placebo mean, (4) prucalopride mean, and (5) mean of all participants. Brain activations showing significant group differences were identified at the whole-brain level using cluster-based thresholding (*Z* > 3.1, familywise error (FWE) *p* < 0.05 corrected). Significant interactions from whole-brain analyses were further explored by extracting percentage BOLD signal change for each type of contrast. As the hippocampus was a particular focus, it was prespecified as a region of interest (ROI). A functional ROI mask was created for the left and right hippocampus by multiplying mean activation for each contrast of interest (on whole-brain data already corrected for multiple comparisons (FWE) and *Z* > 3.1 as described above) for all participants by the Harvard–Oxford subcortical atlas anatomical mask at a 50% threshold. Percentage BOLD signal change for each contrast in each hemisphere was extracted in order to identify the profile of drug effect. All activations are reported using MNI coordinates.

FSLVBM, a voxel-based morphometry style analysis [41], was carried out to investigate potential grey matter differences between the two study groups, underlying and potentially influencing group-related BOLD differences. FSLFIRST [42] was used to segment the left and right hippocampus for each participant, and the volume of individual hippocampi was determined using vertex analysis. This was then normalised for each individual’s brain volume and the resulting values for each hippocampus compared across groups using *t*-tests. Regional and global blood flow was calculated for each individual using the Oxford_ASL [43, 44]. Where appropriate for FSLVBM and ASL, threshold-free cluster enhancement was used to correct for multiple comparisons. Hippocampal perfusion between groups was compared using fslmeants: parameter estimates of perfusion were extracted from resting perfusion maps (previously computed using Oxford_ASL in units of ml/100 g/min) using anatomical Harvard–Oxford masks of the left and right hippocampus at a 50% threshold.

## Results

### Participants

In total, 50 (100% of target) participants were recruited between 11^th^ June 2018 and 17^th^ May 2019. One participant was excluded from all analyses for data-quality concerns raised at the time of data collection. Two other participants were excluded from fMRI analyses at the time of scanning for persistent sleepiness and acute anxiety. A further three participants were excluded from fMRI analyses for (i) a structural brain variant that affected registration to standard space, (ii) a poor-quality structural scan according to MRIQC assessment, and (iii) significant motion during the scan.

Analysis occurred in originally assigned groups. The final groups (*N* = 44, 21:23 = placebo: prucalopride, aged 18–36) were well matched for age, BMI, level of education, use of substances, and NART scores (see Table 1). However, compared to those with English as a first language, for nonnative English speakers, the odds of being in the prucalopride group were 0.17 (95% CI 0.04–0.73, *p* = 0.017). Randomisation guesses (data missing for one participant) suggested that participants were better than chance at guessing group allocation, particularly in the placebo group (correct guess: placebo 75.0%, prucalopride 60.9%). There were no adverse events in the prucalopride group; one person discontinued placebo due to abdominal discomfort.

**Table 1:** Sample demographics, baseline mood & personality.

Variable (measure)	Measurement		Placebo (*N* = 21)	Prucalopride (*N* = 23)	*P* value	Odds ratio (95% CI)
Sex	Number	Female	13	13	Ref	Ref
		Male	8	10	0.77	1.25 (0.37–4.18)^a^
Age	Mean (SD)		24.6 (4.0)	24.7 (5.1)	1.00	
Education level	Number	Postgraduate	8	10	Ref	Ref
		Undergraduate	4	4	0.79	0.8 (0.15–4.25)^b^
		6th form/equivalent	9	9	0.74	0.8 (0.22–3.00)^c^
First language	Number	English	11	20	Ref	Ref
		Not English	10	3	0.02***	0.17 (0.04–0.73)^d^
IQ (NART)	Mean (SD)		112.4 (6.9)	113.4 (5.3)	0.60	
Handedness (EHI)	Mean % right (SD)		84.7 (17.8)	86.5 (22.7)	0.77	
Alcohol	Mean units/week (SD)		2.9 (2.6)	3.8 (3.6)	0.37	
Caffeine	Mean drinks/day (SD)		1.7 (1.1)	1.5 (1.3)	0.63	
Body mass index	Mean (SD)		23.1 (2.8)	23.0 (2.4)	0.85	
Mood (BDI-II)	Mean (SD)		0.9 (1.2)	1.0 (1.2)	0.70	
Anhedonia (SHAPS)	Mean (SD)		0.8 (1.7)	0.6 (1.6)	0.62	
Anxiety (STAI-T)	Mean (SD)		31.6 (7.5)	29.5 (5.4)	0.28	
Personality (EPQ)	Mean (SD)	Neuroticism/Stability	4.6 (3.8)	4.4 (3.1)	0.83	
		Psychotism/Socialisation	2.2 (1.9)	2.1 (1.8)	0.85	
		Extroversion/Introversion	14.9 (3.8)	14.0 (4.3)	0.47	
		Lie/Social desirability	10.6 (3.9)	9.9 (4.3)	0.57	

***<0.05, NART National Adult Reading Test, EHI Edinburgh Handedness Inventory, BDI Beck Depression Inventory, SHAPS Snaith–Hamilton Pleasure Scale, STAI-T Spielberger Anxiety Inventory-Trait, EPQ Eysenck Personality Questionnaire.

^a^OR of being male in prucalopride group, compared to females.

^b^OR of being undergraduate in prucalopride group, compared to postgraduate.

^c^OR of being 6th form in prucalopride group, compared to postgraduate.

^d^OR of being non-English in prucalopride group, compared to English.

### Questionnaire results

There were no significant differences in state anxiety or affect between the prucalopride and placebo group, and no significant differences in reported side effects at baseline or during the study visit (all *p*s = >0.5, see Supplementary Table 1).

### Behavioural results of memory task

Both groups had a high level of accuracy on the prescan task and were able to correctly identify the images as familiar or different with over 95% accuracy and no significant differences between the two groups (*t*(1,42) = −1.323, *p* = 0.193; placebo *M* = 95.72, SD = 13.02; prucalopride *M* = 99.43, SD = 1.84; data missing for three participants (1 from prucalopride, 2 from placebo = 41 participants included)). This suggests that both groups had adequately encoded the prescan images as required.

**Fig. 2:**
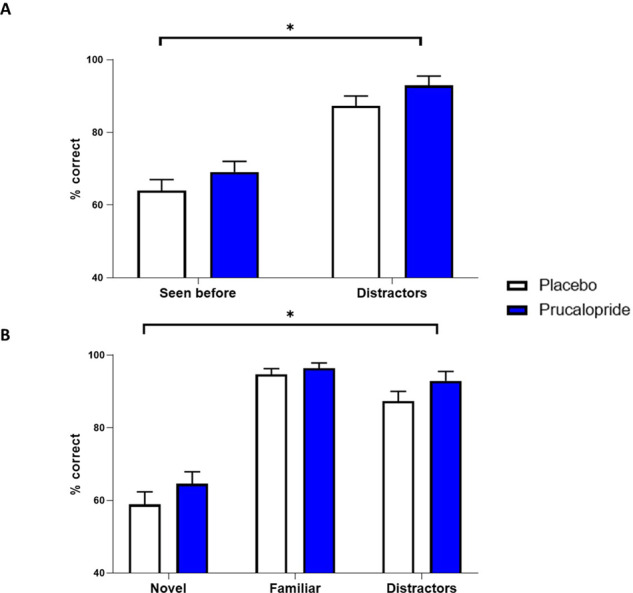
Post-scan recall task results (percentage total correct at identifying image type) divided by group. White = placebo, blue = prucalopride. *p < 0.05, signifying a main effect of group on ANOVA both when comparing placebo vs. prucalopride for **A** seen before (novel + familiar) vs. distractors; **B** novel vs. familiar vs. distractors.

Administration of prucalopride led to improved memory performance in the post-scan recall task: the prucalopride group were more accurate than the placebo group in selecting those seen before (novel + familiar) versus distractors (*F*(1,38) = 5.180, *p* = 0.029, *ηρ*2 = 0.12; prucalopride (*M* = 81.04, SEM = 1.63), placebo (*M* = 75.67, SEM = 1.70)) (see Fig. 2(A)) and also distinguishing each category of image (novel/familiar/distractor) (*F*(1,38) = 4.806, *p* = 0.035, *ηρ*2 = 0.11; prucalopride (*M* = 84.65, SEM = 1.36), placebo (*M* = 80.32, SEM = 1.43)) (see Fig. 2(B)) (data missing for four participants (two from each group = 40 participants included)).

### Memory task fMRI

#### Main effect of task

For all participants, similar to that previously described [30, 31], the novel versus familiar contrast revealed increased BOLD fMRI signal intensity bilaterally in the hippocampus, parahippocampal gyrus, and temporal fusiform cortex. There was also increased activity to novel versus familiar pictures in association areas, including the thalamus, and regions in the frontal (precentral gyrus, cingulate/paracingulate gyrus, superior frontal gyrus, and frontal pole) and occipital lobes, basal ganglia (caudate, putamen), and amygdala (see Supplementary Table 2).

#### Effect of treatment—region-of-interest analysis

**Fig. 3:**
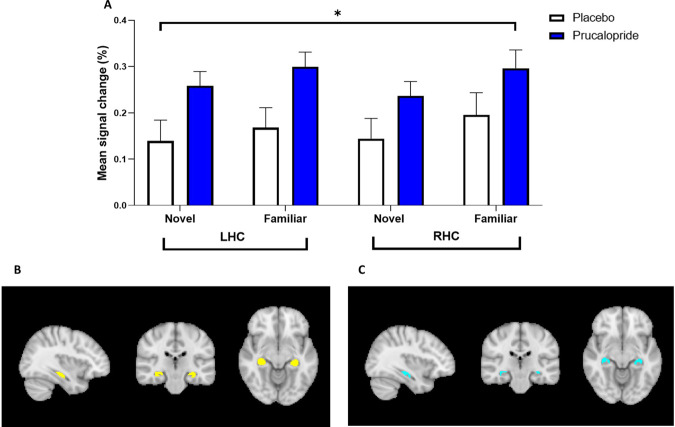
BOLD percentage signal change extracted from functional left (LHC) and right (RHC) hippocampal mask in response to novel and familiar images. **A** Group mean of BOLD percentage signal change extracted from LHC and RHC in response to novel and familiar images. Error bars show standard error of the mean. *p < 0.05, signifying a main effect of group on ANOVA. **B** A functional ROI mask was created for the left and right hippocampus for each contrast of interest by multiplying mean activation for all participants by the anatomical mask at a 50% threshold. LHC and RHC mask in sagittal, coronal, and axial views created for novel mean (yellow); **C** LHC and RHC mask in sagittal, coronal, and axial views created for familiar mean (light blue).

In the left and right hippocampus, there was increased activity in the prucalopride group compared with the placebo group in response to both novel and familiar images (see Fig. 3). There was a significant effect of group [*F*(1,42) = 5.19, *p* = 0.028, *ηρ*2 = 0.11], although there was no significant condition*hemisphere*group interaction present. To understand this main effect of group, we explored each condition and hemisphere separately. When we explored the role of condition, there were significant effects of group for both novel [*F*(1,42) = 3.94, *p* = 0.036, *ηρ*2 = 0.10] and familiar images [*F*(1,42) = 3.09, *p* = 0.027, *ηρ*2 = 0.11].

#### Effect of treatment—whole-brain analysis

**Fig. 4:**
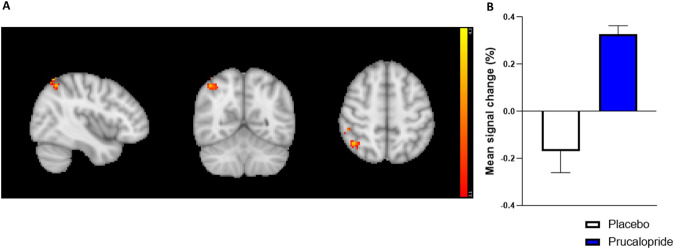
Whole-brain activation in response to familiar mean in prucalopride vs. placebo group. **A** Sagittal, coronal, and axial images depicting significantly increased activation in the prucalopride group for the familiar mean contrast in a right angular gyrus cluster (peak voxel location *x* = 42, *y* = − 60, *z* = 60, *Z* = 4.1, cluster size = 168 voxels). Images thresholded at *z* > 3.1, *p* < 0.05 corrected. Red-to-yellow colours identify increases in brain activation. **B** Group mean of BOLD percentage signal change extracted from the right angular gyrus cluster (familiar mean). Error bars show standard error of the mean. White = placebo, blue = prucalopride.

There was increased activity in the prucalopride group compared with the placebo group in response to familiar images in the right angular gyrus [prucalopride > placebo, Z = 4.1, *p* < 0.005, peak voxel location: *x* = 42, *y* = −60, *z* = 60, cluster size = 168 voxels] (see Fig. 4(A)). Fig. 4(B) represents the group-level-extracted BOLD signal change for this activation cluster.

### Analyses controlling for potential confounds

There were no group-related differences in grey matter between groups (FSLVBM: placebo > prucalopride, *p* = 0.91; prucalopride > placebo, *p* = 0.44) or in the size of hippocampi compared after individually normalising for total brain volume (FSL FIRST vertex: *p* = 0.55 (right hippocampus), *p* = 0.07 (left hippocampus)). There was also no difference between groups in either global blood flow (Oxford_ASL: *p* = 0.73 (grey matter), *p* = 0.65 (white matter)) or regional blood flow (placebo > prucalopride, *p* = 0.70; prucalopride > placebo, *p* = 0.36). Whole-brain and hippocampal ROI results were similar with and without correction for cerebral perfusion and grey matter maps, and sex (see Supplementary Figure 1). Left and right hippocampal perfusion did not differ between groups: smoothed data *p* = 0.79 (left hippocampus), *p* = 0.47 (right hippocampus) (see Table 2). Native language and post-scan recall accuracy were not correlated using linear regression (*R*^2^ = 0.007).

**Table 2:** Left and right hippocampal mean (SD) perfusion (ml/100 g/min).

		Left hippocampus	Right hippocampus
Smoothed (2.12)	Placebo	52.50 (7.46)	53.84 (8.28)
	Prucalopride	51.92 (6.86)	52.17 (6.66)
Unsmoothed	Placebo	53.72 (7.48)	55.60 (8.48)
	Prucalopride	53.23 (7.02)	53.94 (6.87)

## Discussion

The main findings of our study are that six days’ prucalopride administration in young healthy participants improved recall and increased neural activation in the hippocampus and functionally related areas relative to placebo.

Specifically, in the post-scan recall task, participants on prucalopride were better able to distinguish each type of image, as well as demarcate those seen before and/or during the scan from the new distractor images. Preclinical studies have demonstrated that preadministration of 5-HT_4_ agonists before memory encoding improves learning and memory in animals [11, 45, 46], with evidence of effects enduring with repeated administration (14 days) [47]. Previous work in our laboratory provided an initial translation of these results into humans, where healthy volunteers randomised to a single dose of prucalopride showed better recall and recognition of words in a verbal learning and emotional memory task [29]. The current study replicates and extends the evidence in humans of improved memory recall following prucalopride administration.

Consistent with the improved memory recall seen at a behavioural level, the current study also demonstrated an increase in hippocampal activation for both forms of encoded image with prucalopride. Although the function of memory is not specific to one brain region, the hippocampus is known to play a central role, especially when rich mental imagery is involved [48], and it is closely connected to memory-association areas, including the angular gyrus [49, 50]. In addition to direct effects on the serotonergic system, prucalopride appears indirectly to reduce bursts of AMPA-receptor-mediated currents in the hippocampus, altering glutamatergic transmission [51] and thus making it a particularly interesting candidate for cognitive enhancement. GABAergic and glutamatergic neurones are also thought to mediate the increased release of acetylcholine that occurs with 5-HT_4_-receptor agonism (as 5-HT_4_ receptors are not present on cholinergic basal forebrain neurons) [52], which occurs particularly in the hippocampus and is blocked by 5-HT_4_-receptor antagonists [9, 15]. Our study confirms that 5-HT_4_-receptor agonism with prucalopride appears to lead to hippocampal activation in response to a memory stimulus, which is likely associated with behavioural effects related to cognitive performance.

Interestingly, studies using PET imaging have found an inverse correlation between verbal memory scores and 5-HT_4_-receptor binding in the hippocampus [53] and more globally [54]. At first sight, this seems counter to the notion that 5-HT_4_ receptors facilitate hippocampal-dependent memory. However, the authors suggested that this seemingly paradoxical finding might be explained by dependence of 5-HT_4_-receptor availability on endogenous serotonin release. That is, increased 5-HT_4_-receptor binding might be a marker of a lesser degree of intrinsic stimulation of 5-HT_4_ receptors.

Prucalopride also significantly increased activation in a region associated with memory retrieval (the right angular gyrus) during a task where participants had to recall recently encoded information. The right angular gyrus (Brodmann area 39) has potential as a neuroimaging marker of early cognitive impairment [55], related to its close connections with the hippocampus [49] and as a “core hub” within the default-mode network (DMN) [56]. As activity in the angular gyrus at encoding appears to directly correlate with memory-retention ability [50], one suggested function of this region during memory retrieval is to distinguish objects that were or were not embedded as part of a schema during encoding.

Clinically, angular gyri lesions cause language dysfunction, low mood, and poor memory [57] and can mimic dementia or pseudodementia [57, 58]. Similarly, in individuals with mild cognitive impairment (MCI), the right angular gyrus shows significantly decreased activity during resting-state fMRI compared to healthy controls [55, 59]. Therefore, the increased activity seen in the right angular gyrus following prucalopride administration in our study is consistent with the pro-cognitive behavioural effects we observed, and is in keeping with previous evidence of neural representations of cognition and human cognitive impairment. In support of this suggestion, Jin and colleagues found that activity within the right angular gyrus was positively correlated with behavioural memory scores for both delayed recall and learning efficiency [59].

Furthermore, abnormalities in these areas and networks do not appear to be limited to impairments within primary cognitive disorders, but also extend to other psychiatric conditions [60]. For example, grey matter volumes of the right angular gyrus are reduced in those at genetic risk of psychosis who also have currently impaired cognition compared to healthy controls [61], and in first episode psychosis volumetric reduction in hippocampal subfields appears to correlate significantly with 5-HT_4_ receptor density [62]. Importantly for clinical practice, functional connectivity impairments in the regions and network highlighted in our study appear to be amenable to improvement. For example, in patients with MCI, resting state functional connectivity between the hippocampus and right angular gyrus increased following a six-month mind-body exercise-based intervention versus no intervention, alongside improved cognitive performance scores in the intervention group [63].

5-HT_4_-receptor agonism may not facilitate all forms of learning and memory: in our previous study of acute prucalopride administration, there was no effect on working memory or implicit contextual learning [29]. However, these processes involve circuitry external to the hippocampus [64, 65]. The dose of prucalopride (1 mg) used in both our previous single and current subacute dosing studies was chosen following pilot work where there were difficulties tolerating the 2 mg dose, which is the standard dose used in the treatment of constipation. However, it is possible that 1 mg of prucalopride may be suboptimal from the point of view of cognitive enhancement. We currently lack data that could indicate the dose of prucalopride required to produce the most clinically effective activation of 5-HT_4_ receptors in the brain. Use of this low dose may have decreased power in our study, and may explain, for example, why hippocampal activation to the stimulus contrast did not meet significance at the whole-brain level. Due to chance, most participants whose first language was not English were randomised to placebo. However, our memory task was not verbal, and there was no evidence that first language was correlated with post-scan recall task performance.

The hippocampal memory task used in this study is a validated probe of the hippocampus [30, 31], and therefore appropriate for use with an experimental medicine approach to assess therapeutic potential for treatment-related cognitive improvement [66]. However, although our region-of-interest analyses involving the hippocampus demonstrate a difference between the prucalopride and placebo groups in their strength of activation, this difference did not survive correction for multiple fMRI comparisons in our whole-brain analysis. The power to detect any difference at a statistical level may be improved by an increased prucalopride dose or a larger study. Unfortunately, we had to exclude three participants from analyses due to fMRI-analysis concerns in addition to an earlier three excluded at the time of data collection for technical reasons.

Our study holds promise for the future use of 5-HT_4_-receptor agonists in terms of ameliorating some of the cognitive impairments associated with psychiatric disorders. Our study also provides evidence that the pro-cognitive effects of 5-HT_4_-receptor agonism are still apparent following subacute administration; this is consistent with animal studies. Future work could include optimising the dose of prucalopride to ensure maximal effect at the 5-HT_4_ receptor while minimising the side effects. This may involve higher doses alongside peripheral 5-HT_4_-receptor blockade to prevent side effects, or using a novel pharmacological agent where PET studies have indicated the dose required for optimised brain 5-HT_4_-receptor occupancy. It also would be beneficial to delineate further which specific cognitive domains may benefit from 5-HT_4_-receptor agonism to enable precise targeting of deficits as part of a personalised approach to treatment.

## Supplementary information

Supplementary material
