# Supplementary material for: Déjà-vu? Neural and behavioural effects of the 5-HT4 receptor agonist, prucalopride, in a hippocampal-dependent memory task
Source: Transl Psychiatry. 2021 Oct 4;11:497. doi: 10.1038/s41398-021-01568-4 (PMC8488034; doi:10.1038/s41398-021-01568-4)
Supplement: Supplementary file 1 — Supplementary material [file 41398_2021_1568_MOESM1_ESM.docx]

de Cates *et al.*

**Déjà-vu? Neural and behavioural effects of the 5-HT_4_ receptor agonist, prucalopride, in a hippocampal-dependent memory task**

**Supplementary Material**

##### Supplementary Table 1: Questionnaire measures across visits

|  | Placebo mean (SD)  (N=21) | Prucalopride mean (SD) (N=23)^1^ | P value (2dp) |
| --- | --- | --- | --- |
| Spielberger State Anxiety Inventory (STAI-S)  *Baseline*  *Pre-Testing*  *Post-Testing* | 27.8 (8.1)  27.3 (6.0)  30.5 (8.4) | 25.3 (4.7)  29.0 (5.6)  28.7 (6.8) | 0.59 |
| Positive and Negative Affect Scale –  Positive (PANAS-P)  *Baseline*  *Pre-Testing*  *Post-Testing* | 32.6 (5.1)  33.3 (5.6)  31.9 (7.6) | 33.9 (7.0)  32.3 (6.9)  31.4 (8.1) | 0.66 |
| Positive and Negative Affect Scale –  Negative (PANAS-N)  *Baseline*  *Pre-Testing*  *Post-Testing* | 11.6 (2.3)  12.2 (3.1)  12.6 (3.4) | 10.8 (1.3)  11.6 (2.5)  12.1 (3.0) | Included in ANOVA above |
| Visual Analogue Scale (VAS) –  Happy  *Baseline*  *Pre-Testing*  *Post-Testing* | 70.7 (12.4)  72.2 (13.3)  73.9 (14.9) | 72.3 (12.8)  74.6 (13.2)  76.6 (14.2) | 0.80 |
| Visual Analogue Scale (VAS) –  Sad  *Baseline*  *Pre-Testing*  *Post-Testing* | 10.4 (13.2)  11.8 (16.7)  11.8 (16.5) | 6.7 (12.8)  9.1 (15.4)  5.9 (10.0) | Included in ANOVA above |
| Visual Analogue Scale (VAS) –  Hostile  *Baseline*  *Pre-Testing*  *Post-Testing* | 4.7 (14.3)  8.4 (17.3)  7.6 (18.2) | 4.2 (10.6)  3.9 (10.5)  4.3 (9.9) | Included in ANOVA above |
| Visual Analogue Scale (VAS) –  Alert  *Baseline*  *Pre-Testing*  *Post-Testing* | 62.1 (20.3)  62.8 (21.2)  57.1 (25.1) | 70.8 (22.1)  70.0 (14.7)  62.2 (24.2) | Included in ANOVA above |
| Visual Analogue Scale (VAS) –  Anxious  *Baseline*  *Pre-Testing*  *Post-Testing* | 14.9 (19.3)  13.6 (18.4)  10.7 (12.3) | 11.0 (9.6)  14.6 (18.4)  8.3 (12.6) | Included in ANOVA above |
| Visual Analogue Scale (VAS) –  Calm  *Baseline*  *Pre-Testing*  *Post-Testing* | 78.0 (14.5)  79.8 (13.8)  78.3 (17.5) | 76.8 (12.0)  75.9 (13.8)  74.8 (19.1) | Included in ANOVA above |

^1^ results missing in prucalopride group for 1 participant for VAS

Supplementary Table 2: Cortical co-ordinates of maximal activation for clusters on the main effect of task (novel > familiar, mean of all participants)

| **Cluster number** | **Size** | **Z-max** | **p-value** | **Z-max location (MNI)** | **Regions involved in cluster** |
| --- | --- | --- | --- | --- | --- |
| 1 | 51129 | 9.83 | <0.001 | -40,-80,-12 | Including bilateral occipital cortex and pole, Fusiform cortex & gyrus, Temporal gyrus, Parahippocampal gyrus, Hippocampus, Amygdala, Putamen, Caudate, Thalamus |
| 2 | 1430 | 7.06 | <0.001 | 42,10,30 | Right superior, middle and inferior frontal gyrus, precentral gyrus |
| 3 | 332 | 4.92 | <0.001 | 0,42,-24 | Bilateral frontal medial cortex, cingulate / paracingulate gyrus |
| 4 | 321 | 6.15 | <0.001 | 20,-40,-44 | Right cerebellum |
| 5 | 197 | 4.31 | 0.0027 | 38,34,-10 | Right frontal pole, frontal orbital cortex |

Supplementary Figure 1: Whole brain and hippocampal ROI results corrected for gender only, and uncorrected (not corrected for gender, grey matter, or perfusion)

**
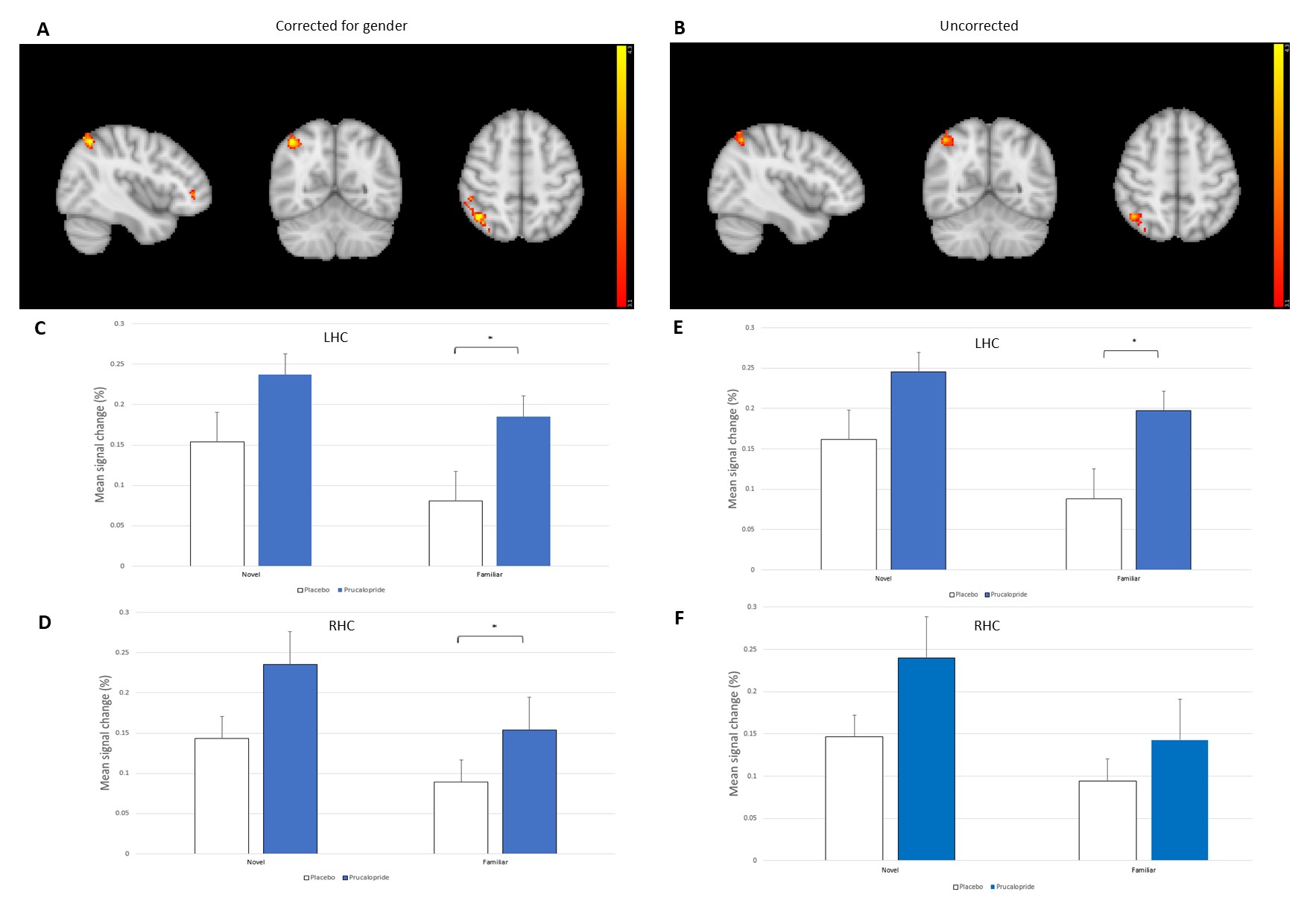
**

*A & B: Whole-brain activation in response to familiar mean in prucalopride vs. placebo group. Sagittal, coronal and axial images depicting significantly increased activation in the prucalopride group for the familiar mean contrast with A: corrected for gender only & B: uncorrected. Activation cluster patterns very similar to corrected for ASL + grey matter maps (as presented in Figure 4(A)). Images thresholded at z> 3.1, p<0.05 corrected. Red to yellow colours identify increases in brain activation.*

*C – F: Group mean of BOLD percentage signal change extracted from functional left hippocampal (C & E) and right hippocampal (D & F) masks in response to novel and familiar images. C & D: corrected for gender only; E & F: uncorrected. Signal change patterns very similar to corrected for ASL + grey matter maps (as presented in Figure 3(A)). Error bars show standard error of the mean.* ** = p<0.05.*

**Additional Supplementary Material**

Participant inclusion and exclusion criteria

| **Inclusion Criteria** |  |
| --- | --- |
| Participant is willing and able to give informed consent for participation in the study  Not currently taking any medications (except the contraceptive pill)  Male or female  Aged 18-40 years  Sufficiently fluent English to understand and complete the task  Right handed  Body Mass Index in the range of 18-30 |  |
|  |  |
| **Exclusion criteria**  Not fluent in English  Any past or current Axis 1 DSM-V psychiatric disorder  Current usage of psychoactive medication (except the contraceptive pill, the Depo-Provera injection or the progesterone implant)  Current usage of any medication that will influence the MRI scan |  |
| Current or past history of drug or alcohol dependency  Currently pregnant or breastfeeding  Study visits due to take place during the pre-menstrual week (female participants will be asked details of their menstrual cycle to schedule the study outside this week)  Not right handed  Body Mass Index outside the range of 18-30  History of cardiac, thyroid, or liver problems  An autoimmune disorder  Current, or a history of, gastro-intestinal disorder or irritable bowel syndrome  Epilepsy  Known lactate deficiency or any other problem absorbing lactose, galactose, or glucose  Participation in a study which uses the same computer tasks as those used in the present study  Participation in a study that involves the use of a medication within the last three months  Smoker > 5 cigarettes per day  Typically drinks > 6 caffeinated drinks per day  Any contraindication to MRI scanning (e.g. metal objects in your body, pacemakers, significant claustrophobia) | |

Full details of fMRI data acquisition and analysis

**MRI data acquisition**

fMRI acquisition: Encoding memory was assessed from a single run of 60 T2-weighted echoplanar imaging (EPI) slices covering the whole brain [repetition time (TR) 800 ms, echo time (TE) 30 ms, flip angle 52°, field of view 216 mm, slice thickness 2.4mm, voxel dimension 2.4mm isotropic, acquisition time 6min 48s]. Images were distortion corrected by an acquired fieldmap (echos at 4.92 and 7.38 ms, TR=590ms, flip angle = 46°).

Structural MRI acquisition: Additional high-resolution T1-weighted structural scans were acquired using a gradient echo sequence (TR 1900ms, TE 3.97ms, flip angle 8°, field of view 192mm, voxel dimension 1 mm isotropic, acquisition time 5min 31s) to allow later registration of the fMRI data into standard space.

ASL acquisition: Each participant also had a resting state pCASL perfusion-weighted scan with a 2D gradient spin echo readout and a PICORE Q2T labelling scheme. ASL data were collected as tag-control pairs with a TI of 1.8 seconds and a bolus duration of 0.7 seconds. ASL imaging parameters were: repetition time: 4100ms; minimum echo time: 14.0ms; FOV read: 220mm; FOV phase: 100%; voxel size: 3.4x3.4x4.5mm; 24 slices with 4.5mm thickness; echo spacing: 0.56mm; EPI factor: 64; post-labelling delays at: 250ms, 500ms, 750ms, 1000ms, 1250ms, and 1500ms; number of dynamics/repeats: 97 (1 volume was control); acquisition time = 6min 39s; fat saturation = on. A calibration image was acquired without labelling (TR = 6000ms).

Labelling plane was set with a time of flight neck scan (TR = 21ms, TE = 3.43ms, flip angle = 30°, field of view = 200mm, voxel dimension = 0.3 x 0.3 x 1.3 mm, acquisition time = 42s). Images were distortion corrected by an acquired fieldmap (echos at 4.92 and 7.38ms, TR=482ms, flip angle = 46°).

**MRI analysis**

fMRI task analysis: Imaging data were analysed with FSL ([www.fmrib.ox.ac.uk/fsl](http://www.fmrib.ox.ac.uk/fsl)).

fMRI data were pre-processed and analysed using FEAT (FMRI Expert Analysis Tool), version 6.0.4, part of FSL (FMRIB’s Software Library; www.fmrib.ox.ac.uk/fsl). DICOM (Digital Imaging and Communications in Medicine) files were downloaded from the server, checked for completeness, excessive movement and visual anomalies, and converted to a BIDS (Brain Imaging Data Structure)-standardised format nifti files using heudiconv (heudiconv 0.5.4 (<https://github.com/nipy/heudiconv)>) before pre-processing. The structural anatomical scans were brain extracted using the Brain Extraction Tool (BET)^1^. Formal MRI quality assessment was undertaken using the MRIQC package (<https://mriqc.readthedocs.io/en/stable/index.html>), with data considered for rejection if it fell outside the normal range of values in the derived image quality metrics.

Pre-processing involved: motion correction using FMRIB’s Linear Image Registration Tool (FLIRT^2^); deletion of non-brain tissue using BET ^1^; spatial smoothing with a Gaussian kernel of 5 mm full-width-half-maximum; grand-mean intensity normalisation of the entire 4D dataset by a single multiplicative factor; high pass temporal filtering (Gaussian-weighted least-squares straight line fitting, with sigma of 90s) and B0 unwarping using fieldmap phase and magnitude images for distortion correction. No slice timing correction was applied. In addition, registration to high-resolution image and to a standard template [Montreal Neurological Institute (MNI)] was implemented using FNIRT nonlinear registration ^3^.

In the first-level analysis, individual activation maps were computed using the general linear model with local autocorrelation correction. Two explanatory variables were modelled: “novel” and “familiar” images. Temporal derivatives were included in the model. Variables were modelled by convolving each block with a haemodynamic response function, using a variant of a gamma function (standard deviation 3s, mean lag 6s). No included participant demonstrated significant movement: absolute displacements were less than 1 voxel and relative displacements less than ½ voxel. At the whole-brain level, familiar images were contrasted with novel, resulting in the following model: 1) novel vs. baseline; 2) familiar vs. baseline; 3) novel > familiar; 4) novel < familiar.

In the second-level analysis, whole-brain individual data were combined at a group level (participants on placebo vs. prucalopride) using a mixed-effects analysis , and cerebral blood flow and grey matter maps as covariates of no interest. Groups were contrasted with each other, resulting in the following comparisons: 1) placebo > prucalopride; 2) prucalopride > placebo; 3) placebo mean; 4) prucalopride mean; 5) mean of all participants. Brain activations showing significant group differences were identified at the whole-brain level using cluster-based thresholding (Z>3.1, family-wise error (FWE) p<0.05 corrected). Significant interactions from whole-brain analyses were further explored by extracting percentage BOLD signal change for each type of contrast. As the hippocampus was a particular focus, it was pre-specified as a region of interest (ROI). A functional ROI mask was created for the left and right hippocampus by multiplying mean activation for each contrast of interest (on whole-brain data already corrected for multiple comparisons (FWE) and Z>3.1 as described above) for all participants by the Harvard-Oxford subcortical atlas anatomical mask at a 50% threshold. Percentage BOLD signal change for each contrast in each hemisphere was extracted in order to identify the profile of drug effect. All activations are reported using MNI co-ordinates.

FSLVBM, a voxel-based morphometry style analysis ^4^, was carried out to investigate potential grey matter differences between the two study-groups, underlying and potentially influencing group-related BOLD differences. Brain-extracted images (automatically created for each individual using FSLanat) were tissue-type segmented. Grey matter partial volume images were aligned to standard space using FLIRT and FNIRT registration tools. The resulting images were averaged, modulated and smoothed with an isotropic Gaussian kernel of 2mm to create a study-specific-template. A voxel-wise GLM was then applied using permutation non-parametric testing (5000 permutations). FSLFIRST ^5^ was used to segment the left and right hippocampus for each participant, and the volume of individual hippocampi were determined using vertex analysis. This was then normalised for each individual’s brain volume and the resulting values for each hippocampus compared across groups using *t*-tests.

Regional and global blood flow was calculated for each individual. Distortion and motion corrected resting perfusion maps in units of mL/100g/min were calculated using Oxford_ASL (part of the Bayesian Inference for Arterial Spin Labelling (BASIL) tool, https://fsl.fmrib.ox.ac.uk/fsl/fslwiki/BASIL; ^6, 7^ for each participant, which performs label-control subtraction, inference of voxelwise perfusion, and voxelwise calibration to obtain absolute perfusion maps, and controls for partial volume effects at the single subject level. FSL’s Anatomical Processing Script (FSL_Anat, https://fsl.fmrib.ox.ac.uk/fsl/fslwiki/fsl_anat) was used to pre-process each participant’s high resolution T1 structural image (includes bias-field correction, brain extraction and registration to standard space via FMRIB’s Linear Image Registration Tool (FLIRT) and FMRIB’s Non-linear Image Registration Tool (FNIRT). The processed perfusion images were non-linearly aligned with standard space via an initial linear transformation T1 structural space (using FLIRT), followed by application of the non-linear warp from fsl_anat. A Gaussian smoothing kernel of 2.12mm was applied to all the normalised images (to match functional data). Data were interrogated using voxel-wise generalized linear model (GLM) permutation nonparametric testing (5,000 permutations) with randomise (FSL’s tool for nonparametric permutation inference on neuroimaging data), correcting for multiple comparisons across space (cluster-based thresholding using TFCE and a family-wise error (FWE)-corrected cluster significance threshold of p<0.05 applied to the suprathreshold clusters). This results in spatial maps characterising the between-subject/group differences.

As post-hoc analyses, whole brain group feat results were also run using cerebral blood flow and grey matter maps as covariates of no interest, and sex as a covariate of interest. Hippocampal perfusion between groups was compared using fslmeants: parameter estimates of perfusion were extracted from resting perfusion maps (previously computed using Oxford_ASL in units of ml/100g/min) using anatomical Harvard-Oxford masks of the left and right hippocampus at a 50% threshold.

References for supplementary material

1. Smith SM. Fast robust automated brain extraction. *Hum Brain Mapp* 2002; **17**(3)**:** 143-155.

2. Jenkinson M, Bannister P, Brady M, Smith S. Improved optimization for the robust and accurate linear registration and motion correction of brain images. *Neuroimage* 2002; **17**(2)**:** 825-841.

3. Andersson J, Jenkinson M, Smith S. Non-linear registration, aka Spatial normalisation. FMRIB technical report TR07JA22007.

4. Douaud G, Smith S, Jenkinson M, Behrens T, Johansen-Berg H, Vickers J *et al.* Anatomically related grey and white matter abnormalities in adolescent-onset schizophrenia. *Brain* 2007; **130**(Pt 9)**:** 2375-2386.

5. Patenaude B, Smith SM, Kennedy DN, Jenkinson M. A Bayesian model of shape and appearance for subcortical brain segmentation. *Neuroimage* 2011; **56**(3)**:** 907-922.

6. Chappell MA, Groves AR, Whitcher B, Woolrich MW. Variational Bayesian inference for a nonlinear forward model. *Trans Sig Proc* 2009; **57**(1)**:** 223-236.

7. Chappell MA, Groves AR, MacIntosh BJ, Donahue MJ, Jezzard P, Woolrich MW. Partial volume correction of multiple inversion time arterial spin labeling MRI data. *Magn Reson Med* 2011; **65**(4)**:** 1173-1183.
